# TRUNK BODY MASS INDEX: A NEW REFERENCE FOR THE ASSESSMENT OF BODY MASS DISTRIBUTION

**DOI:** 10.1590/0102-672020180001e1362

**Published:** 2018-06-21

**Authors:** Mariane TAKESIAN, Marco Aurelio SANTO, Alexandre Vieira GADDUCCI, Gabriela Correia de Faria SANTARÉM, Julia GREVE, Paulo Roberto SILVA, Roberto de CLEVA

**Affiliations:** 1 Department of Gastroenterology; 2 Department of Orthopedics and Traumatology, Medical School of University of São Paulo São Paulo, SP, Brazil.

**Keywords:** Body composition, Body mass index, Fat mass, Severe obesity, Trunk height., Composição corporal, Índice de massa corporal, Massa de gordura, Obesidade severa, Altura do tronco

## Abstract

**Background:**

Body mass index (BMI) has some limitations for nutritional diagnosis since it does not represent an accurate measure of body fat and it is unable to identify predominant fat distribution.

**Aim:**

To develop a BMI based on the ratio of trunk mass and height.

**Methods:**

Fifty-seven patients in preoperative evaluation to bariatric surgery were evaluated. The preoperative anthropometric evaluation assessed weight, height and BMI. The body composition was evaluated by bioimpedance, obtaining the trunk fat free mass and fat mass, and trunk height. Trunk BMI (tBMI) was calculated by the sum of the measurements of the trunk fat free mass (tFFM) and trunk fat mass (tFM) in kg, divided by the trunk height squared (m^2)^). The calculation of the trunk fat BMI (tfBMI) was calculated by tFM, in kg, divided by the trunk height squared (m^2)^). For the correction and adjustment of the tBMI and tfBMI, it was calculated the relation between trunk extension and height, multiplying by the obtained indexes.

**Results:**

The mean data was: weight 125.3±19.5 kg, height 1.63±0.1 m, BMI was 47±5 kg/m^2)^ and trunk height was 0.52±0,1 m, tFFM was 29.05±4,8 kg, tFM was 27.2±3.7 kg, trunk mass index was 66.6±10.3 kg/m², and trunk fat was 32.3±5.8 kg/m². In 93% of the patients there was an increase in obesity class using the tBMI. In patients with grade III obesity the tBMI reclassified to super obesity in 72% of patients and to super-super obesity in 24% of the patients.

**Conclusion:**

The trunk BMI is simple and allows a new reference for the evaluation of the body mass distribution, and therefore a new reclassification of the obesity class, evidencing the severity of obesity in a more objectively way.

## RESUMO

**Racional:**

O índice de massa corporal (IMC) para diagnóstico nutricional apresenta limitações, pois não representa medida precisa da adiposidade corporal, podendo assim subestimar a presença de obesidade.

**Objetivo:**

Desenvolver um índice de massa corporal baseado entre a relação da massa e altura do tronco.

**Método:**

Cinquenta e sete pacientes em preparo pré-operatório para cirurgia bariátrica foram submetidos à avaliação antropométrica (peso, altura e índice de massa corporal). Para cálculo do IMC do tronco foi avaliada a composição corporal pela bioimpedância, obtendo-se a massa livre de gordura e massa de gordura do tronco; a medida do tronco foi calculada pela diferença entre a altura a partir da sétima vértebra cervical e a extensão dos membros inferiores. O cálculo do IMC do tronco (IMCt) foi a soma das medidas da massa livre de gordura do tronco (MLGt) e massa de gordura do tronco (MGt), em kg, dividindo-se pelo quadrado da altura do tronco (m^2)^). O IMC de gordura do tronco (IMCgt) foi calculado utilizando a MGt, em kg, dividindo-a pelo quadrado da altura do tronco (m^2)^). Para correção e ajuste do IMCt e IMCgt foi calculada a relação entre os valores de extensão do tronco e da altura, multiplicando-se pelo valor dos índices obtidos.

**Resultados:**

As médias do peso e altura foram de 125,3±19,5 kg e 1,63 m±0,1, respectivamente, e do IMC de 47±5 kg/m^2)^. A média da altura do tronco foi de 0,52±0,1 m, da MLGt de 29,05±4,8 kg, da MGt de 27,2±3,7 kg, do IMCt de 66,6±10,3 kg/m², e do IMCgt 32,3±5,8 kg/m². Em 93% dos pacientes houve aumento da classificação da gravidade da obesidade com o cálculo do IMCt . Nos pacientes com obesidade grau III, o IMCt alterou a classificação para super-obesidade em 72% dos pacientes e para super-super obesidade em 24% dos pacientes.

**Conclusão:**

O IMC do tronco é método antropométrico acessível e prático, que permite a reclassificação do IMC baseado na distribuição da massa do tronco, evidenciando de forma mais clara a gravidade da obesidade.

## INTRODUCTION

The World Health Organization (WHO) considers obesity as the greatest threat to public health nowadays. Approximately 400 million adults are obese and 1.6 billion overweight^4^ ^,^ ^15^. 

Obesity is a multifactorial disease, mainly characterized by excessive body fat related to the development of important comorbidities such as type 2 diabetes mellitus, dyslipidemia, cardiovascular disease, arterial hypertension and metabolic syndrome^1^ ^,^ ^8^ ^,^ ^12^ ^,^ ^15^ ^,^ ^25^_._ Severe obesity is characterized by excessive body fat, increased total body water and reduced lean mass^3^.

Body mass index (BMI) is an anthropometric method developed in 1832 by the mathematician Adolphe Quételet, aiming to determine the ideal body mass of an individual^26^. WHO recommends BMI in the diagnose of obesity^27^. However, in recent years, there has been increasing debate about the development of different BMI cutoffs for different ethnic groups, due to the growing evidence that associations between BMI, body fat percentage and body fat distribution differ between populations and, therefore, the health risks increase below the 30 kg/m^2 )^cutoff^15^, that defines obesity in the current WHO classification^2^ ^,^ ^27^.

Additionally, the use of BMI for nutritional diagnosis has limitations, once it does not represent a precise measure of body adiposity, being unable to differentiate fat free mass from fat mass^4^ ^,^ ^5^ ^,^ ^13^ ^,^ ^23^, and may underestimate the presence of obesity in about 40% of cases^6^.

With the increasing importance of the diagnosis of obesity, it is necessary to re-evaluate the way in which body fat and its distribution are determined^23^, since health risk is different depending on the location of the fat accumulation (i.e. in the upper or lower half of the body)^8^ ^,^ ^28^. The distribution of body fat, specifically visceral (or central) fat, seems to be the link between adipose tissue and insulin resistance, characteristic of metabolic syndrome. It is questioned the exclusive use of BMI in the classification of cardiovascular risk in obese individuals, taking into consideration that even populations with low BMI present a high prevalence of metabolic syndrome^18^.

Visceral fat is more associated with metabolic complications than abdominal and peripheral subcutaneous fat. Men present, on average, 20% or more of total fat as visceral fat, while women, less than 10%^16^. Thus, it is believed that an index based on the disposition of central body fat may be more specific to diagnose the obesity severity, due to the relevance of intra-abdominal adipose tissue, currently considered a multiple-function organ^18^.

The aim of this study was to propose a body mass index based on the relationship between trunk mass and height.

## METHODS

All participants signed the informed consent form. The study protocol was approved by the Ethics Committee from the Clinics Hospital from University of São Paulo (number 01038912.6.0000.0068).

A total of 77 patients were selected from January to October 2016, aged 18-60 years and BMI between 40-60 kg/m^2^, admitted to the Bariatric and Metabolic Surgery Unit of the Discipline of Digestive Tract Surgery, Clinics Hospital, Medicine School, University of São Paulo, São Paulo, SP, Brazil.

Twenty patients were excluded due to acute or chronic disease that caused excessive water retention (n=2), patients bedridden or with functional limitation (n=16) or previous bariatric surgery (n=2).

### Anthropometric and body composition

Participants were weighed in light clothing, without carrying heavy objects, in orthostatic position, with the lower limbs parallel, without footwear, eyes straight ahead, with the upper limbs at the side of the body and without moving, at the center of a microelectronic scale installed on the smooth surface to avoid oscillation (InBody 230®, GE Healthcare, USA with 250kg capacity, with 100g intervals). The height was measured with the feet and heels parallel, shoulders and glutes leaning against the portable stadiometer graduated in millimeters (Sanny®, American Medical do Brasil Ltda). BMI was calculated using the cutoff points suggested by WHO^2^ ^,^ ^26^ ^,^ ^27^.

Body composition was assessed using a noninvasive indirect bioimpedance method (InBody 230®), where the participant was positioned in orthostatic position, without moving or talking, on a platform with special supports for the bare feet (lower electrodes) and with the upper limbs extended holding two supports with the hands (upper electrodes). The scale used directly measured the impedance of each body segment at 20KHz and 100KHz sampling frequency, leading to highly accurate results. The chemical composition of the body fat free mass (FFM) was conventionally assumed to be constant, with a density of 1.1 kg/m^3^, with a temperature of 37° C and a water concentration of 73%. Thus, FFM of the upper limbs, trunk and lower limbs were calculated by multiplying the water volume of the upper extremities (the sum of the right and left), trunk, and lower limbs (the sum of the right and left extremities) by 1.37^21^ .

The following data were obtained: trunk FFM (FFMt) and trunk fat mass (FMt), in absolute values ​​and percentage.

To obtain the trunk height, the patients remained with parallel lower limbs, without footwear. They were measured using an inelastic measuring tape with a two meter length. It was measured, by physical examination, the distance from the seventh cervical vertebra (C7), located in the back of the neck, to the floor and from the iliac crest (located at the back of the hip, by physical examination) to the floor, subtracting the second from the first measure, obtaining the trunk height.

The trunk BMI (BMIt) was calculated using the sum of the FFMt and trunk fat mass (FMt), in kg, divided by the square of the trunk height (m^2^).

The trunk fat BMI (BMIft) was calculated using FMt, in kg, by dividing it by the square of trunk height (m^2^).

### Indexes correction factor

To correct and adjust the BMIt and BMIft indexes, the relationship between the trunk extension and the height was calculated, multiplying by the values obtained: a) BMIt correction factor: trunk (m)/height (m) x BMIt; b) BMIft correction factor: trunk (m)/height (m) x BMIft

## RESULTS

The sample consisted of 57 patients (39 women). The mean weight and height were 125.3±19.5 and 1.63±0.09 kg, respectively. BMI showed an average of 47±5 kg/m^2^.

The mean patient´s trunk extension was 0.52 m, being 0.56 m in men and 0.49 m in women. The mean FFMt was 29.1 kg and the mean FMt was 27.2 kg (Table 1).

**TABLE 1:** Anthropometric values and trunk body composition

Variables (n=57)	Mean	± SD	Minimum	Maximum
Trunk (m)	0.52	± 0.1	0.29	0.68
Trunk FFM (kg)	29.1	± 4.8	20.3	43.5
Trunk FM (kg)	27.2	± 3.7	17.3	34.7
Trunk FFM + FM (kg)	56.2	± 7.7	38.7	75.1

FFM=fat free mass; FM=fat mass

The results of the BMI are shown in Table 2. Applying the index correction factor, the mean corrected BMIt was 66.62 kg/m² and 32.32 kg/m² for the corrected BMIft.

**TABLE 2:** Body mass indexes

BMI (n=57)	Mean	± SD	Minimum	Maximum
BMI (kg/m²)	47.0	± 5.0	39.4	58.9
Trunk BMI (kg/m²)	216.1	± 54.4	144.0	350.0
Corrected Trunk BMI (kg/m²)	66.6	± 10.3	36.5	87.0
Trunk fat BMI (kg/m²)	104.9	± 27.9	62.1	174.4
Corrected Trunk Fat BMI (kg/m²)	32.3	± 5.8	18.8	44.2

BMI=body mass index

Out of the 39 patients with obesity class III, BMIt reclassified 37 patients (95%): nine (24%) were classified as super obese (BMI>50 kg/m²) and 28 (72%) as super-super obese (BMI>60 kg/m²).

Out of the 16 super obese patients (28%), BMIt reclassified 13 patients (81%) as super-super obese. 

Only four patients maintained the obesity classification after usinf BMIt, and only one reduced the obesity classification (Table 3).

**TABLE 3:** Patients body mass index reclassification after applying the correction factor

Patients (n=57)	Mean BMI (kg/m²)	Classification	Mean correctecd BMIt (kg/m²)	Reclassification
13	53.15	super obese	69.95	super super obese
3	54.65	super obese	55.68	super obese
28	45.25	class III	71.57	super super obese
9	43.58	class III	56.06	super obese
1	43.69	Class III	48.87	class III
1	48.06	class III	36.52	class II
2	39.68	class II	63.55	super super obese

BMI=body mass index; BMIt=trunk body mass index

## DISCUSSION

One of the main objectives of determining body composition is to estimate the amount of body fat, related to the presence of systemic diseases, morbidity and mortality^17^ ^,^ ^22^. It should be emphasized that the simple measurement of body mass is not able to identify the lack or excess of body components (fat mass, muscle mass, water, and bone mass)^2^.

BMI is recommended for its convenience, safety and simplicity^10^. However, there are important limitations related to interpretations that should be considered, such as age, gender, and ethnicity^12^ ^,^ ^20^. In addition, BMI does not estimate body fat mass, thus limiting its accuracy in the diagnosis of obesity^20^ ^,^ ^22^.

There is no consensus on the best method for body evaluation in patients with severe obesity^2^. In the literature, some studies suggest new parameters for the evaluation of body composition and classification of nutritional status by body fat percentage, such as body fat index (BFI), which uses only anthropometric measures such as hip circumference and stature^5^ ^,^ ^13^ ^,^ ^29^ and reciprocal ponderal index (RPI) in which are considered the individual height and weight^7^.

BFI is a fast, inexpensive and non-invasive method that was developed with Mexican American adults to estimate percentage of body fat^5^. Studies have shown that its performance was not consistent in other populations with different characteristics from those used in its development and validation^4^. For Chinese population BFI underestimated the percentage of body fat when compared to bone densitometry measured by DEXA (dual energy X-ray absorptiometry). Thus, BFI is not better as an indicator of cardiovascular risk compared to BMI^29^.

In a study with 102 Brazilian women with BMI 26.9±3.1 kg/m^2^, the percentage of body fat obtained by DEXA and BFI were compared, with averages 36.9% and 33.6%, respectively. BFI showed low agreement and accuracy, varying according to age, gender and ethnicity^6^. A study with 433 patients with severe obesity compared BFI and noninvasive indirect bioimpedance with an already validated equation adapted for this population to calculate the percentage of fat, and found limitations in BFI method for this population^3^.

Reciprocal ponderal index (RPI)^22^, calculated by the equation: height (cm)/weight (kg)^1^ ^-^ ^3^, has as cutoff points >44: underweight, 41-44: normal and <41: overweight. It shows greater mathematical logic and less influence of extreme height, since weight is a cubic variable and height is linear, when compared to BMI^19^. The use of RPI, which also does not discriminate fat free mass and fat mass, would, theoretically, have the same limitations as BMI^7^ ^,^ ^19^. However, when it is used for the diagnosis of overweight and obesity, compared to the percentage of body fat the result is lower, especially in women, and also, RPI is directly influenced by age and gender^7^.

A study with 530 Japanese Brazilians with a prevalence of overweight and central adiposity (ratio between waist and hip circumference) of 22% and 67%, respectively, showed that individuals with central adiposity had higher glycemia, triglycerides, total cholesterol, low-density lipoprotein and lower high-density lipoprotein rates compared to those without overweight and central adiposity^14^.

A review with 433 articles showed that non-invasive indirect bioimpedance (with specific equations) and BFI are inexpensive and non-invasive methods that are available and can be routinely used to estimate body fat^2^.

Central obesity, characterized by the accumulation of trunk and abdomen fat, has as one of its components, visceral abdominal fat, which its thickness is of great importance as an indicator of cardiovascular risk^9^.

 The development of an index that is more objectively evidence the relation of trunk mass and fat distribution can contribute to obesity classification and its relationship with associated diseases. In this study 96% of the obese patients class III had increased obesity class (for super and super-super obesity), demonstrating the usefulness of using trunk BMI as a new proposal for the evaluation of obese patients in bariatric surgery programming.

## CONCLUSION

The trunk body mass index is an accessible and practical anthropometric method, which allows the reclassification of BMI based on trunk mass distribution, evidencing more clearly the severity of obesity.

## References

[1] Barros F, Setúbal S, Martinho JM, Ferraz L, Gaudêncio A (2016). Correlation of non-alcoholic fatty liver disease and features of metabolic syndrome in morbidly obese patients in the preoperative assessment for bariatric surgery. ABCD, arq. bras. cir. dig.

[2] Belarmino G, Horie LM, Sala PC, Torrinhas RS, Heymsfield SB, Waitzberg DL (2015). Body adiposity performance in estimating body fat in a sample of severely obese Brazilian patients. Nutrition Journal.

[3] Bergman RN, Stefanovski D, Buchanan TA, Sumner AE, Reynolds JC, Sebring NG, Xiang AH, Watanabe RM (1083). A Better index of body adiposity. Obesity, 2011;19(5);.

[4] Bernhard AB, Santo MA, Scabim VM, Serafim MP, de Cleva R (2016). Body Composition Evaluation in Severe Obesity A Critical Review. Advances in Obesity, Weight Management & Control.

[5] Bernhard AB, Scabim VM, Serafim MP, Gadducci AV, Santo M.A., de Cleva R (2017). Modified body adiposity index for body fat estimation in severe obesity. Journal of Human Nutrition and Dietetics.

[6] Cerqueira M, Amorim P, Magalhães F, Castro E, Franco F, Cerqueira SFL, Marins J, Doimo L (2013). Validity of body adiposity índex in predicting body fat in sample of. Brazilian women.Obesity.

[7] Damasceno VO, Dutra LN, Ribeiro LG, Vianna VRA, Vianna JM, Novaes JS, Lima JRP (2003). Índice de Massa Corporal e Recíproco do índice ponderal na identificação de sobrepeso e obesidade. Revista Brasileira de Cineantoprometria & Desempenho Humano.

[8] Diniz MTC, Diniz MFHS, Almeida SR, Savassi-Rocha AL, Ferreira JT, Savassi-Rocha PR (2003). Tratamento cirúrgico da obesidade mórbida em mulheres do tipo androide e ginecoide: estudo prospectivo e comparativo. Arquivo Brasileiro Cirurgia Digestiva.

[9] Gouvêa HR, Faria SL, Faria OP, Cardeal MA, Bezerra A, Ito MK (2013). Validação da ultrassonografia para a avaliação da gordura abdominal visceral em obesos clinicamente graves. Arquivo Brasileiro Cirurgia Digestiva.

[10] Holanda LGM, Carvalho MMC, Souza MD, Carvalho CMRG, Assis RC, Leal LMM, Mesquita LPL, Costa EM (2011). Excesso de peso e adiposidade central em adultos de Teresina-PI. Revista de Associação Médica Brasileira.

[11] Moore Keith L, Dalley Arthur E (2001). Anatomia orientada para a clínica.

[12] Kopelman PG (2000). Obesity as a medical problem. Nature.

[13] Lam BCC, Lim SC, Wong MTK, Shum E, Ho CY, Bosco JIE, Chen C, Koh GCH (2013). A Method Comparison Study to Validate a Novel Parameter of Obesity, the Body Adiposity Index, in Chinese Subjects. Obesity Biology and Integrated physiology.

[14] Lerario DDG, Gimeno SG, Franco LJ, Lunes M, Ferreira SRG, Grupo de estudos de Diabetes da Comunidade Nipo-Brasileira (2002). Excesso de Peso e gordura abdominal para a síndrome metabólica em nipo-brasileiros Revista de Saúde. Publica.

[15] Nora C, Morais T, Nora M, Coutinho J, Carmo I, Monteiro MP (2016). Gastrectomia vertical e bypass gástrico no tratamento da síndrome metabólica. Revista Portuguesa de Endocrinologia, Diabetes e Metabolismo.

[16] Oliveira CL, Mello MT, Cintra IP, Fisberg M (2004). Obesidade e síndrome metabólica na infância e adolescência. Revista de Nutrição.

[17] Pinto AS, Chedid MF, Guerra LT, Álvares-da-Silva MR, Araújo A, Guimarães LS, Leipnitz I, Chedid AD, Kruel CRP, Grezzana-Filho TJM, Kruel CDP (2016). Estimating basal energy expenditure in liver transplant recipients: The value of the harris-benedicts equation. ABCD, arq. bras. cir. dig.

[18] Ribeiro FF, Mariosa LS, Ferreira SRG, Zanella MT (2006). Gordura Visceral e Síndrome Metabólica: Mais Que Uma Simples Associação. Arquivo Brasileiro Endocrinologia e Metabolismo.

[19] Ricardo RJ, Araujo CGS (2002). Índice de massa corporal Um questionamento cientifica baseado em evidencias. Arquivo Brasileiro de Cardiologia.

[20] Romero-Corral A, Somers VK, Sierra-Johnson J, Thomas RJ, Collazo-Clavelli ML, Korinek J, Allison TG, Batsis JA, Sert-Kuniyoshi FH, Lopez-Jimenez F (2008). Accuracy of body mass index in diagnosing obesity in the adult general population. International journal of obesity.

[21] Santarém GCF, de Cleva R, Santo MA, Bernhard AB, Gadducci AV, Greve JMA, Silva PRS (2015). Correlation between body composition and walking capacity in severe obesity. PLOS ONE.

[22] Serafim PM (2016). Dieta de muito baixo valor calórico em obesos mórbidos no período pré-operatório de cirurgia bariátrica: análise de alteração da composição corporal durante perda de peso aguda.

[23] Shah NR, Braverman ER (2012). Measuring Adiposity in Patients: The Utility of body mass index (BMI), percent body fat, and leptin. PLos ONE.

[24] Smalley KJ, Knerr NA, Kendrick ZV, Colliver JA, Owen OE (1990). Reassessment of body mass indices. The American journal of clinical nutrition.

[25] Souza LBR, Pernambuco LA, Santos MM, Silva JCV (2015). Vocal Complaint, Auditory-Perceptual Assessment of Voice and Vocal Self-Assessment in Women With Morbid Obesity. ABCD, arq. bras. cir. dig.

[26] Westphal P, Ferreira C, Adamczeski M, Camargo L, Santos R, Massaneiro AN, Griten Yabu V, Cordova M, Ribas M, Riske J, Cássia J, Rikowski L, Souza WC (2016). Relação entre índice de massa corporal de Quetelet e o de Trefethen. Revista CPAQV. Centro de Pesquisas Avançadas em Qualidade de Vida.

[27] World Health Organization Obesity:Global database on Body Mass Index.

[28] WRZESINSKI A, Côrrea JM, Fernandes TMB, Monteiro LF, Trevisol FS, Nascimento RR (2015). Complications requiring hospital management after bariatric surgery. ABCD, arq. bras. cir. dig.

[29] Zhang ZQ, Liu YH, Xu Y, Dau W, Su YX, Chen YM (2014). The validity of the body índex in predicting percentage body fat and cardiovascular risk factors among Chinese. Clinical Endocrinology.

